# Avoiding unnecessary intervention in pregnancy: an ultrasound-based decision algorithm for acute flank pain

**DOI:** 10.1186/s12884-026-08823-w

**Published:** 2026-03-13

**Authors:** Ashraf Talaat Youssef, Maged Salah Eldien Elkady

**Affiliations:** 1 Radiology Department, Faculty of Medicine, Fayoum University, Fayoum city, Egypt; 2 Department of Obstetrics and Gynecology, Faculty of Medicine, Misr University for Science and Technology, Cairo city, Egypt

**Keywords:** Renal colic, Pregnancy complications, Ultrasonography, Doppler, Hydronephrosis, Urolithiasis

## Abstract

**Background:**

Acute flank pain during pregnancy presents a diagnostic challenge due to physiological hydronephrosis and the need to avoid ionizing radiation. Clinicians require reliable, non-invasive imaging strategies that guide management and minimize unnecessary intervention.

**Objective:**

To evaluate high-resolution ultrasonography, including Doppler resistive index (RI) measurement, as a structured decision tool for differentiating etiologies of acute flank pain during pregnancy and guiding management.

**Methods:**

Retrospective cohort study including 87 pregnant women presenting with acute flank pain between May 2022 and December 2023.Patients underwent detailed clinical, laboratory, and high-resolution US evaluation: B-mode grading of hydronephrosis, renal parenchymal and perinephric assessment, ureteric and bladder evaluation, and intrarenal arterial Doppler RI measurement. Clinical parameters including pain severity, fever, and inflammatory markers (CRP, WBC) were integrated to support imaging findings Ultrasonographic findings were correlated with clinical course and management outcomes.

**Results:**

Hydronephrosis was detected in 96.5% of patients, predominantly right-sided (77%). Hydroureteronephrosis with Collecting system wall thickening and edema suggestive of inflammation was the most common etiology (75%). Urolithiasis was detected in 23% of patients, high grade obstruction confirmed in 11.5%. Patients with high grade obstruction had elevated intrarenal RI (≥ 0.72; mean 0.78), whereas those with physiological ureteral compression had normal RI with preserved ureteric jets. Conservative management succeeded in 91% of cases; 9% required surgical intervention.

**Conclusion:**

High-resolution US combined with Doppler RI provides a safe, reproducible, and management-oriented decision algorithm for acute flank pain in pregnancy. It differentiates physiological from pathological obstruction, supports conservative management in most patients, and identifies those requiring timely intervention.

## Introduction

Acute renal pain, commonly known as renal colic, represents a critical abdominal emergency during gestation, frequently necessitating prompt diagnosis and management due to the significant potential for severe maternal and fetal adverse effects. Potential complications include preterm delivery, premature rupture of membranes, maternal systemic sepsis, and acute renal injury [1]. 

The gravid uterus induces substantial physiological and anatomical changes in the urinary tract, making pregnant women highly susceptible to dilation. Physiological hydroureteronephrosis is a common finding, reported in 43% to 100% of pregnant women [2]. This dilation typically affects the right side in approximately 85% of patients, attributed to the gravid uterus compressing the right ureter at the pelvic brim [3]. This process often begins around 20 weeks of gestation and progresses noticeably during the third trimester [4].

Accurate diagnosis requires the integration of clinical features, laboratory investigations, and appropriate imaging. Ultrasonography (US) remains the diagnostic imaging modality of choice due to its non-invasiveness, safety (avoidance of ionizing radiation), high availability, and cost-effectiveness. Conventional radiography and Computed Tomography (CT) pose risks of ionizing radiation exposure and fetal hazards [4, 5]. Magnetic Resonance Imaging (MRI), while useful, is less readily available, more expensive, and has been reported to have lower sensitivity for detecting small stones [5].

 Ultrasonography is pivotal in delineating the cause of acute flank pain, allowing for differentiation between obstructive (calculi) and non-obstructive (physiological) etiologies. US can accurately localize the site of obstruction and estimate stone size [6, 7]. Crucially, the estimation of the average Resistive Index (RI) in the intra-renal interlobular arteries using color Doppler US is a valuable technique to further distinguish between high-grade acute obstruction and non-obstructive uropathy, often showing a marked increase in the RI of the obstructed kidney [8–10].

 Management of this condition hinges on precise diagnosis and necessitates close, collaborative care between the obstetrician and urologist. The clinical goals are dual: preserving renal function and preventing obstetric complications [11].

## Aim of the work

*T*o evaluate the prevalence of various etiologies of acute flank pain in pregnancy and assess the utility of high-resolution US, including Doppler RI, as the foundation for a structured decision algorithm guiding conservative versus interventional management.

## Methodology

### Study design and patients

This retrospective cohort review included 87 consecutive pregnant females presenting with acute renal pain who were admitted to the obstetric department of a large private hospital. Patient ages ranged from 18 to 38 years (mean age: 28 years), and gestational ages spanned 7 to 35 weeks (mean gestational age: 23 weeks). The study period was from May 2022 to December 2023. We acknowledge that as a retrospective study, there is an inherent risk of selection bias, as patients requiring intervention may have had more severe clinical presentations initially.

### Clinical and laboratory assessment

All patients underwent a thorough clinical examination and a standard panel of laboratory tests, including complete blood picture (CBP), C-reactive protein (CRP), erythrocyte sedimentation rate (ESR), urinalysis and culture, and serum urea and creatinine levels. Clinicians prioritized red flags, including fever, signs of systemic inflammatory response (SIRS), and pain refractory to standard analgesia.

### Ultrasonographic examination

All patients underwent combined abdominal and obstetric ultrasound examinations using a SonoAce X8 ultrasound machine (Medison, Korea)*.*

#### Obstetric US

Performed to confirm fetal viability, number of fetuses, gestational age, placental location, and amniotic fluid volume.

#### Abdominal US (Non-obstetric)

Performed to screen for other non-renal causes of acute abdominal pain (e.g., cholelithiasis, appendicitis). Both kidneys and the bladder were meticulously examined.

####  Hydronephrosis grading

The degree of hydronephrosis was graded on B-mode imaging as per standard criteria [4]: Grade I (minimal dilation), Grade II (mildly dilated renal pelvis with non-dilated calyces), Grade III (moderately dilated renal pelvis with dilated calyces), and Grade IV (markedly dilated renal pelvis and calyces with attenuated parenchymal thickness).

#### Parenchymal & perinephric assessment

Detailed B-mode assessment included the thickness of the mucosal layer in the pelvicalyceal system (P/C), the turbidity of urine contents (suggestive of inflammation). Collecting system inflammation was diagnosed based on hypoechoic mucosal thickening of the renal pelvis > 2 mm. The echogenicity of the renal parenchyma, and the echogenicity of the perinephric fat planes were assessed (suggestive of inflammation /abscess/ pyelonephritis).

####  Doppler studies

The average intrarenal arterial Resistive Index (RI) was estimated using a color duplex evaluation. The pulsed Doppler signal was obtained from the interlobar or interlobular arteries at the upper, middle, and lower poles of the kidney, and an average value was calculated to help distinguish true obstruction from physiological dilation [8–10]. While we utilized an absolute RI cutoff of 0.72 as an exploratory threshold, the inter-renal RI difference was monitored to improve diagnostic precision [8].

#### Ureteral & bladder assessment

The ureters were evaluated for dilation, compression, the presence of obstruction-causing stones, and the site and size of the stones. The urinary bladder wall thickness, presence of vesical stones. Ureteric jets were evaluated using power Doppler over a 5-minute observation period to ensure a dynamic assessment of ureteral patency and symmetry of color flow jets from the ureteric orifices. Cystitis was defined by a bladder wall thickness > 4 mm in a moderately distended bladder with visible internal debris.

In our cohort, high-resolution US findings were sufficient for clinical decision-making, and no patients required additional MRI evaluation.

### Data analysis

Clinical, laboratory, and US results were analysed. Follow-up records were reviewed to assess the effectiveness of medical versus surgical management.

## Results

A total of 87 pregnant women presented with acute flank pain. The distribution by trimester was: First Trimester (8%, *n* = 7), Second Trimester (63%, *n* = 55), and Third Trimester (29%, *n* = 25).

### Ultrasonographic findings and etiology

**Fig. 1:**
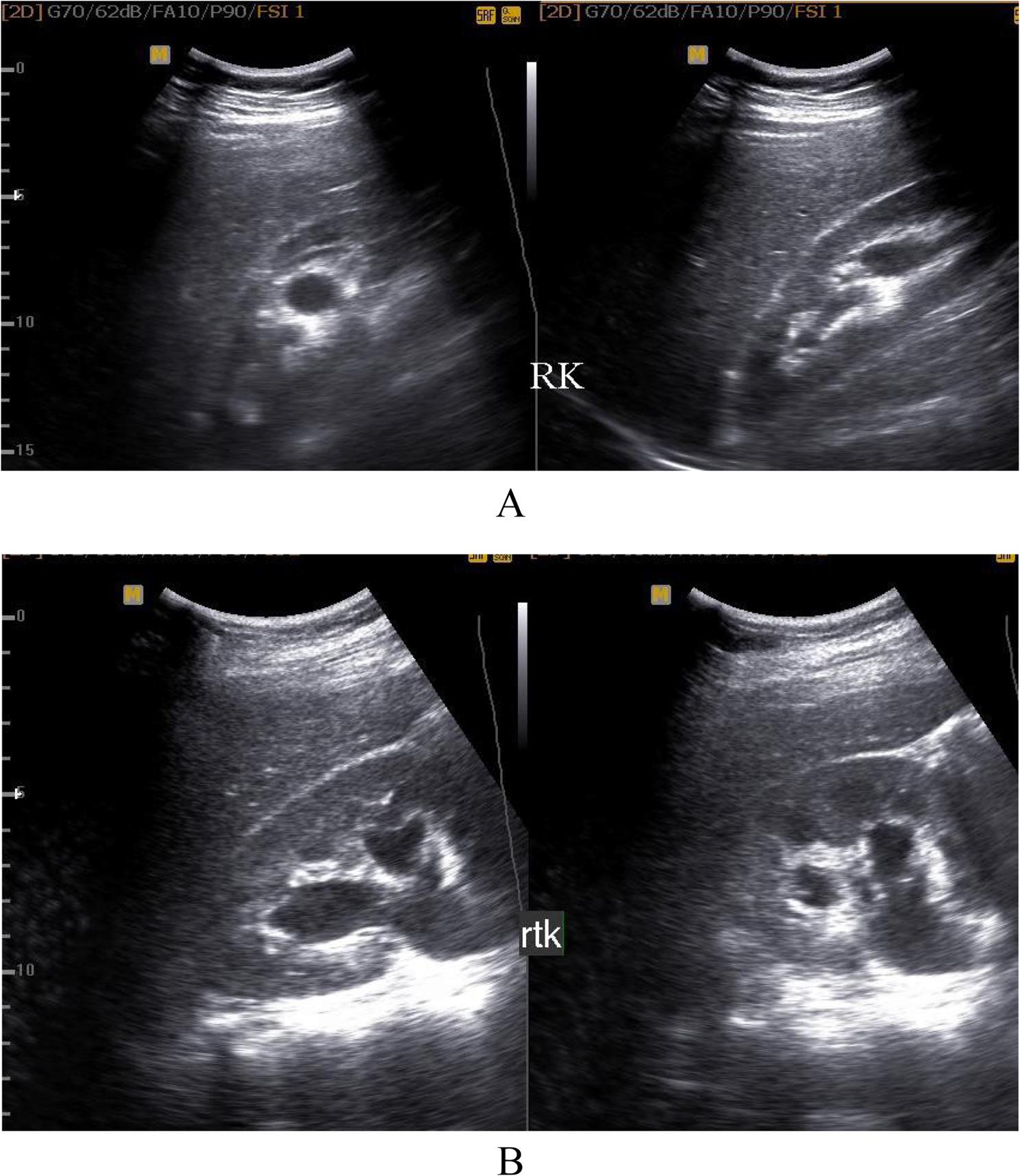
**A**, **B**: B-mode ultrasound exam showing right-grade 11 hydronephrosis (**A**) and another case of right-grade 111 hydronephrosis (**B**)

**Fig. 2:**
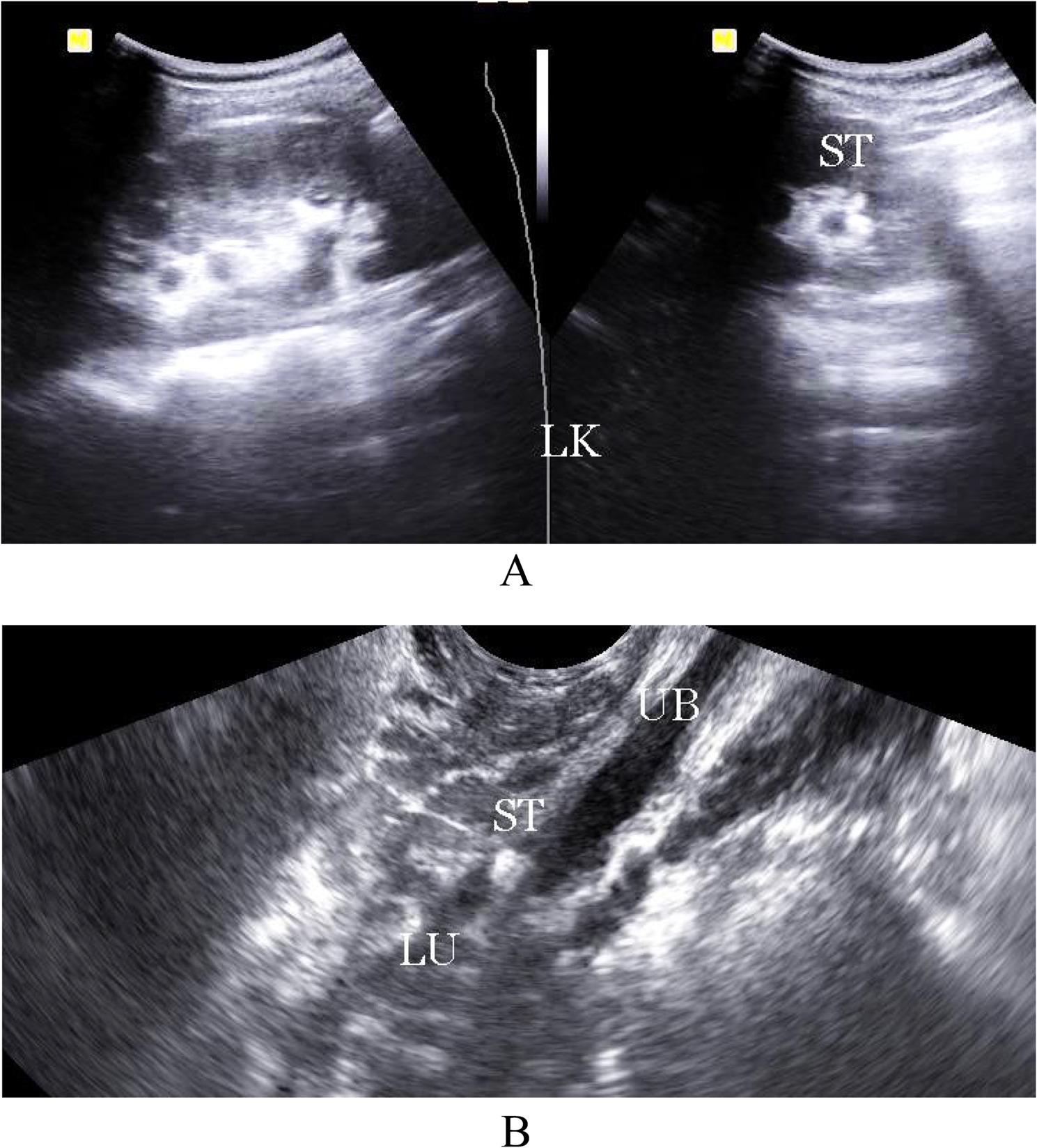
**A**, **B**: B-mode ultrasound examination showing left-grade 11 hydronephrosis with a small calyceal stone (**A**) subsequent to the left terminal ureteric small stone (ST) at the intramural course of the left ureter (LU)

**Fig. 3:**
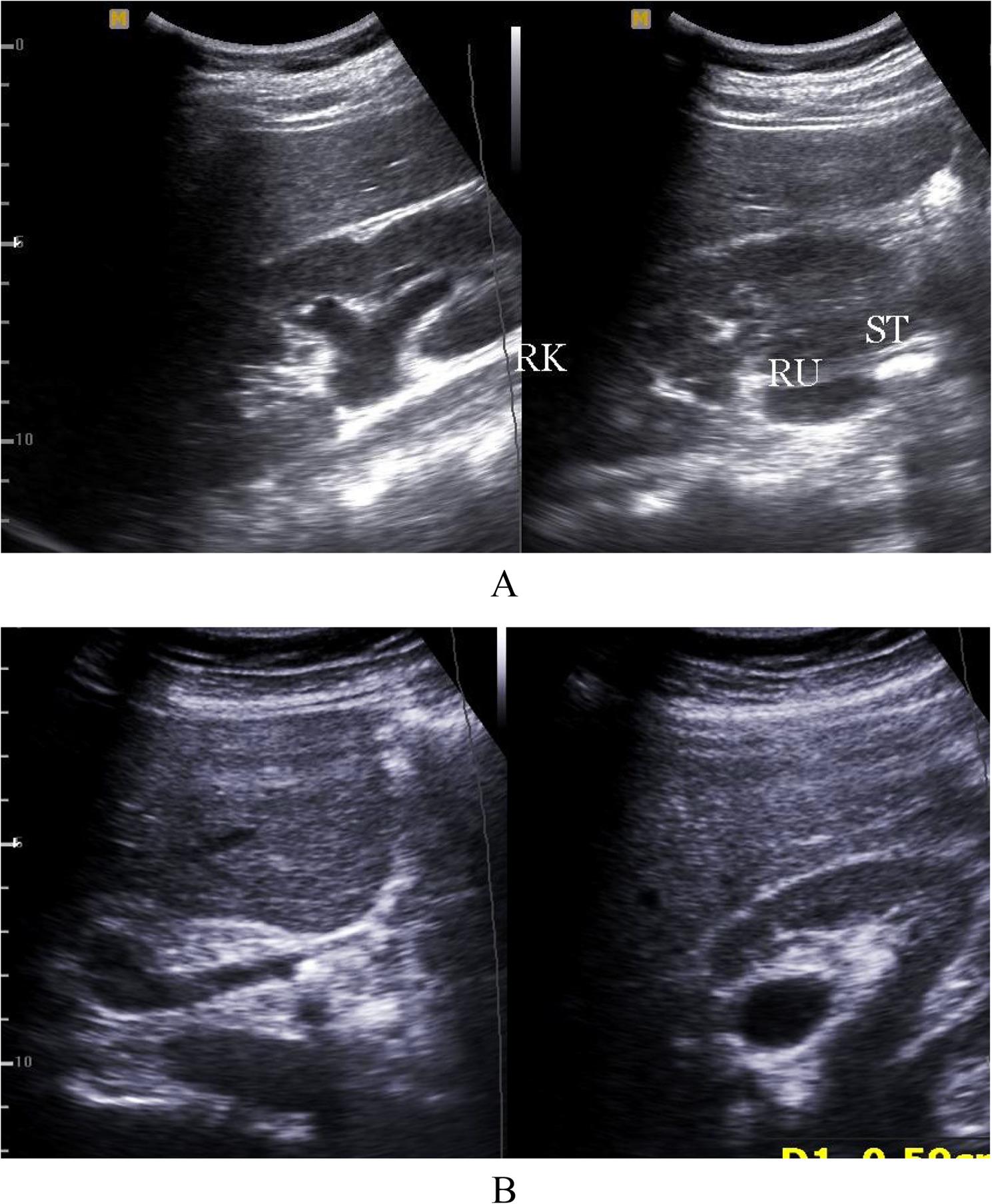
**A**, **B**: B-mode ultrasound examination showing a large stone at the right renal pelviureteric junction (**A**) and a small stone in the proximal course of the right ureter (**B**)

**Fig. 4:**
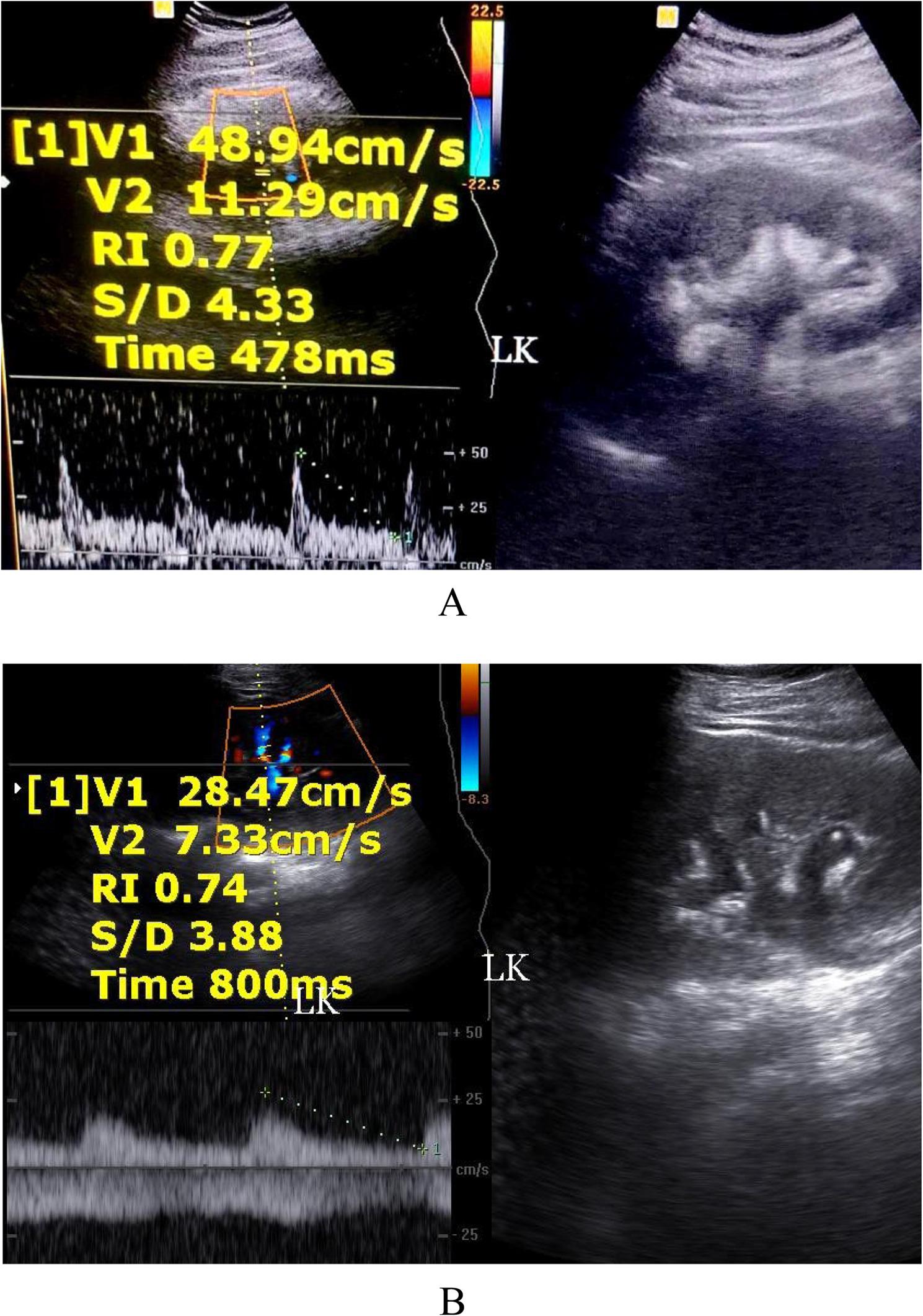
**A**, **B**: Color duplex examination of the left kidney in 2 patients showing obstructive hydronephrosis with an elevated intrarenal interlobar artery resistive index

**Fig. 5:**
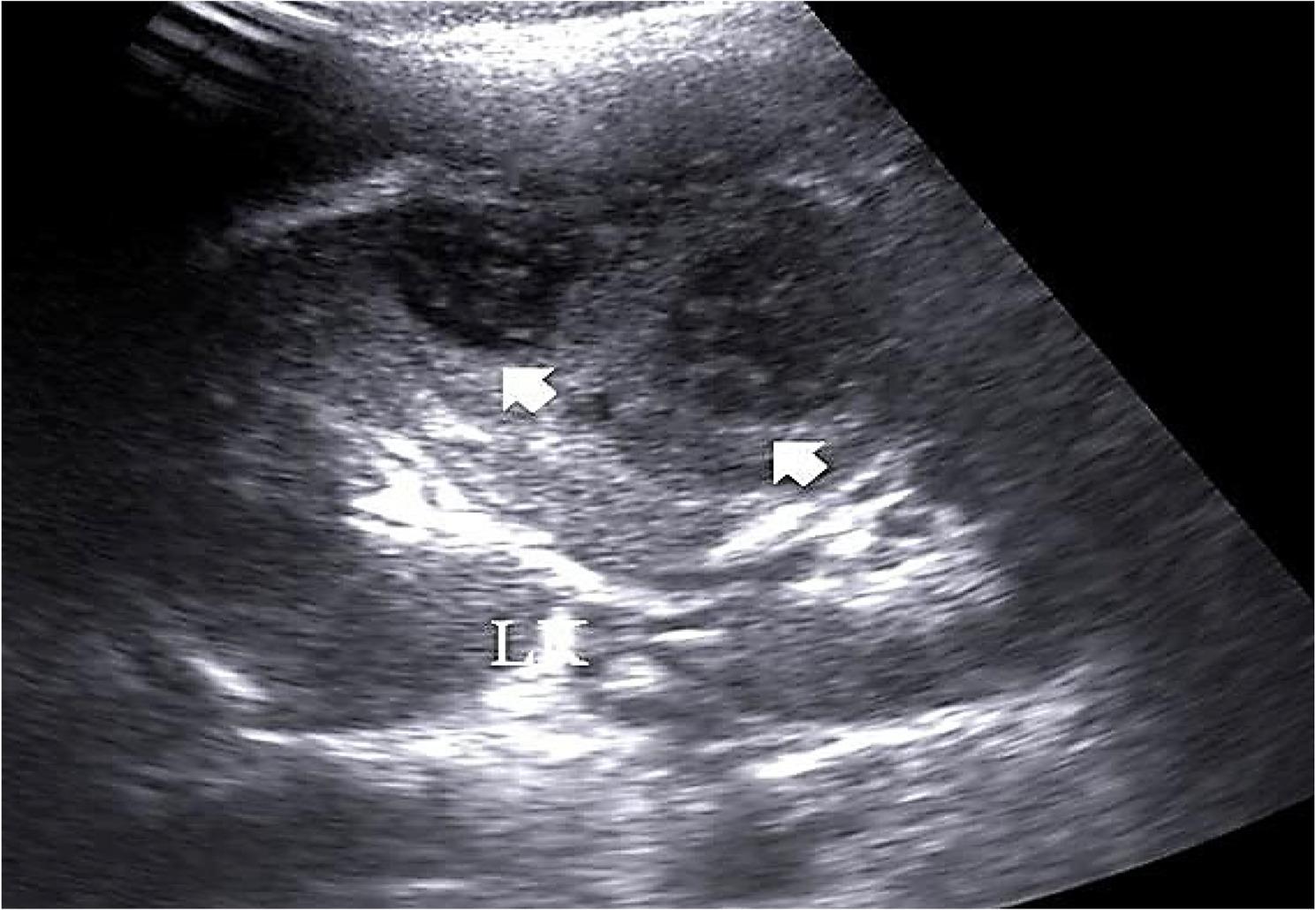
B-mode ultrasound of the left kidney (LK) showing 2 cysts with thick walls and turbid fluid contents suggesting renal abscesses (arrows)

**Fig. 6:**
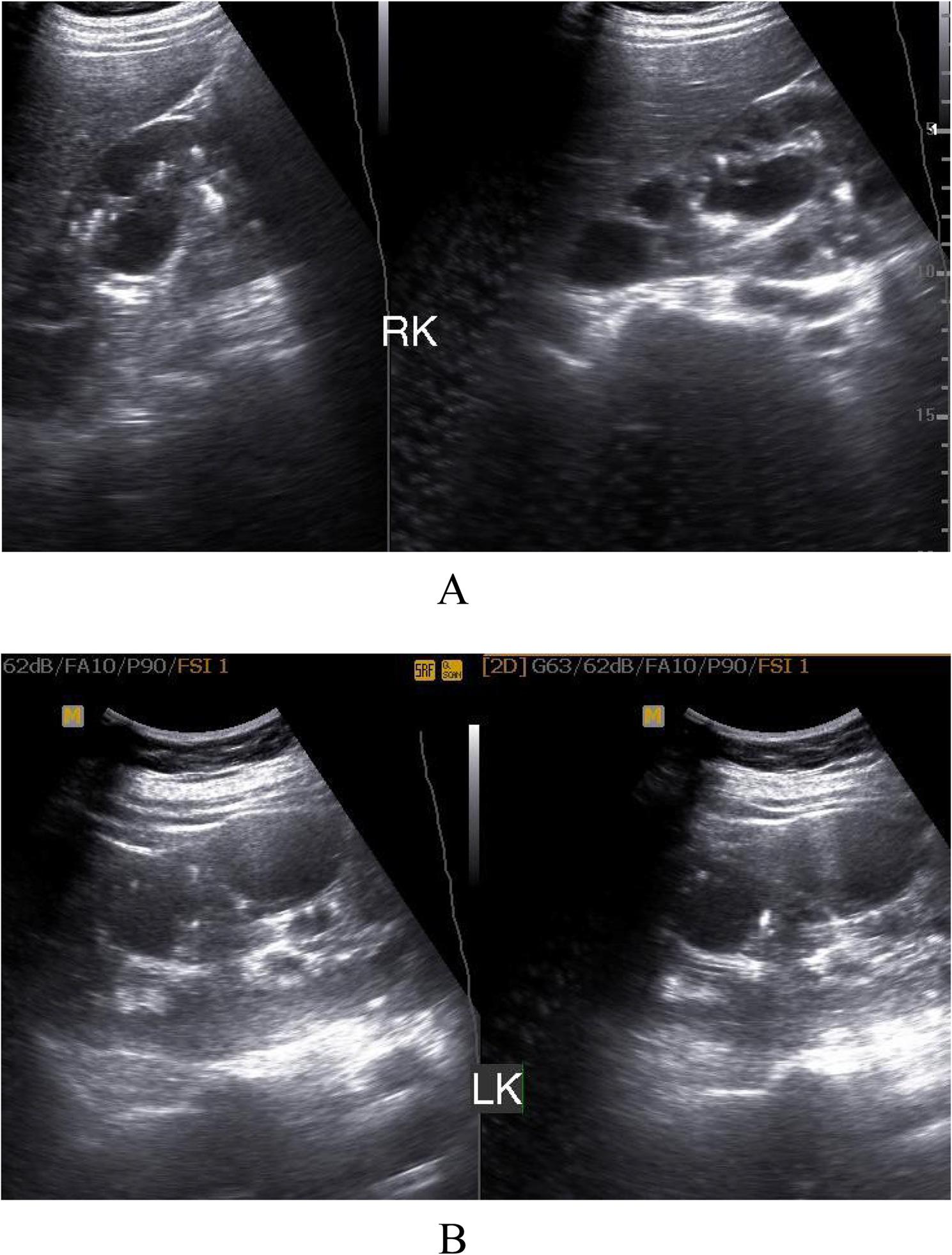
**A**, **B**: B-mode ultrasound examination showing bilateral multiple renal parenchymal cysts suggesting adult polycystic kidney disease with an associated bilateral multiple renal sandy stone

- Hydronephrosis: Eighty-four patients (96.5%) presented with hydronephrotic changes; only 3 patients showed no pelvicalyceal system dilation. The distribution of hydronephrosis severity was: Grade I (4.5%, *n* = 4), Grade II (8%, *n* = 7), Grade III (55%, *n* = 48), and Grade IV (17%, *n* = 15) (Fig. 1A, B).
- Laterality: Hydronephrosis was unilateral on the right side in 77% (*n* = 65), the left side in 12% (*n* = 10), and bilateral in 11% (*n* = 9).
- Inflammation **(**Collecting system wall thickening and edema ): Right renal pyelitis (Pelvicalyceal mucosal wall thickening and turbid urine) was present in 63% (*n* = 55), left renal pyelitis in 11.5% (*n* = 10), and no pyelitis in 25% (*n* = 22) (Table 1). Cystitis was found in 25% (*n* = 22).
- Urolithiasis: Urinary tract stones were detected in 23% (*n* = 20). High grade obstruction secondary to ureteric stones was confirmed in 11.5% (*n* = 10), including 6 terminal ureteric stones (Fig. 2A, B) and 4 pelviureteric junction /upper 1/3 ureteric stones (Fig. 3A, B). Five patients had ureteric stone sizes exceeding 7 mm.
- Resistive Index: The intrarenal arterial Doppler RI was elevated (RI ≥ 0.72) in all 10 obstructed patients, with a mean RI of 0.78 (range 0.72 to 0.84) (Fig. 4A, B). Patients with ureteral dilation attributed to uterine compression had normal RI values.
- Other Findings: Incidental findings included 7 patients with ectopic intrapelvic kidneys (one obstructed by a pelviureteric junction stone). Acute pyelonephritis with no back-pressure changes was detected in 3 patients, among them one patient at 7 weeks had 2 left renal abscesses, (Fig. 5). Adult polycystic kidney disease was detected in 1 patient (Fig. 6A, B).

**Table 1:** Prevalence and laterality of hydronephrosis and associated pyelitis in pregnant patients (*N* = 87)

Laterality/Diagnosis	Number of Patients (*n *= 87)	Percentage (%)
Hydronephrotic Changes (*n* = 84)		
Right-Sided Unilateral Involvement	65	77%
Left-Sided Unilateral Involvement	10	12%
Bilateral Involvement	9	11%
Associated Pyelitis		
Right Renal Pyelitis	55	63%
Left Renal Pyelitis	10	11.5%
No Pyelitis/Pure Hydronephrosis	22	25%

### Patient management outcome

Overall, 91% (*n* = 79) of cases were managed medically (analgesics, antibiotics) and expectantly with an excellent outcome. Only 9% (*n* = 8) necessitated surgical intervention, which included: 5 patients with stones > 7 mm, 2 patients with Grade IV hydronephrosis (managed with ureteric stenting), and 1 patient with renal abscesses (managed by US-guided percutaneous aspiration) (Table 2). Decisions for surgery were based on a multidisciplinary assessment of clinical failure—specifically refractory pain or infection—coupled with high-risk US findings (stones > 7 mm, Grade IV hydronephrosis, or renal abscess).

**Table 2:** Management outcomes

Management Type	Number of Cases (*n* = 87)	Outcome Percentage (%)
Conservative/Expectant (Medical)	79	91%
Surgical Intervention	8	9%
Surgical Indications (*n* = 8):		
Stones > 7 mm: 5 cases		
Grade IV Hydronephrosis (Stenting): 2 cases		
Renal Abscesses (Aspiration): 1 case		

### Ultrasound-based decision algorithm (Illustrating Fig. 7)

**Fig. 7:**
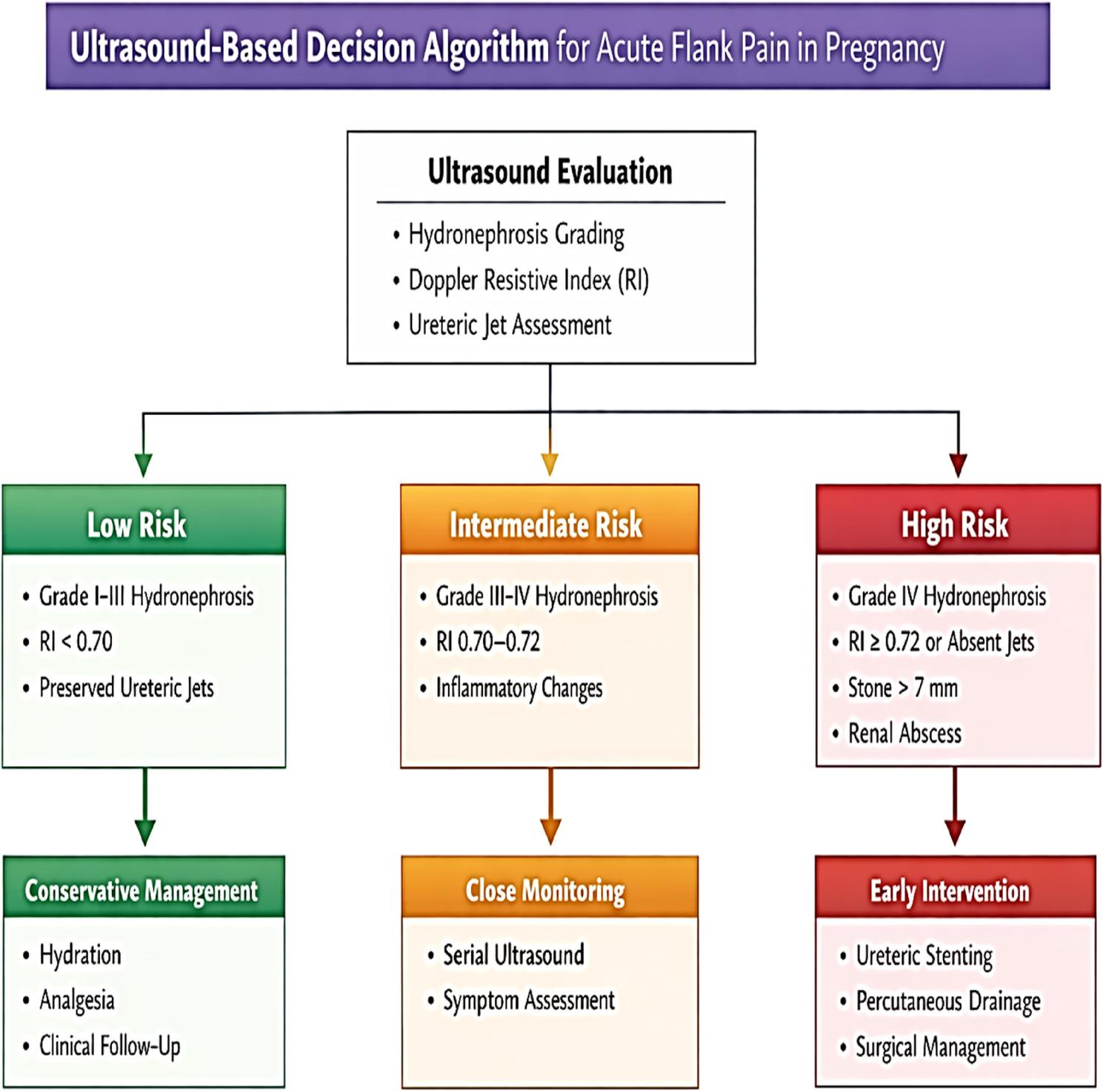
The ultrasound-based decision algorithm for acute flank pain

- Low-risk: Hydronephrosis ≤ Grade III, RI < 0.70, preserved ureteric jets → conservative.
- Intermediate-risk: Grade III–IV hydronephrosis with RI 0.70–0.72 or inflammatory changes → close monitoring.
- High-risk: Grade IV, hydronephrosis, RI ≥ 0.72, absent jets, stones >7 mm, renal abscess → early surgical/interventional management.

## Discussion

This study highlights that while the majority of pregnant patients presenting with acute flank pain demonstrate hydronephrosis (96.5%), the underlying etiology is diverse and requires accurate non-invasive distinction to guide therapy. Our finding that hydroureteronephrosis with secondary collecting system inflammation (75%) was the most prevalent specific diagnosis among hydronephrotic kidneys underscores the high risk of ascending infection secondary to urinary stasis [12].

Ultrasonography is essential for a comprehensive evaluation, providing: the degree of hydronephrotic changes predictive of severity, assessment of parenchymal changes (e.g., echogenicity) suggesting pyelonephritis, and visualization of the ureter down to the site of obstruction, differentiating external compression (gravid uterus) from intrinsic obstruction (calculus) [4, 5].

### The diagnostic value of the resistive index

 A key finding in this cohort was the diagnostic utility of the intrarenal RI. The RI was significantly elevated ( ≥ 0.72) in all cases of high grade obstructive uropathy, confirming its high predictive value for true, pathological obstruction. Conversely, patients whose ureteral dilation was attributable to physiological uterine compression showed a normal RI and symmetric ureteral jets on power Doppler. The ability of Doppler US to reliably differentiate these two groups is critical, as it supports conservative management for physiological cases while flagging obstructive cases for prompt intervention [8–10]. While this study utilizes an absolute threshold of 0.72, literature suggests the inter-renal RI difference is a vital marker for improving accuracy in unilateral obstruction [8].

### Laterality and etiology

The high incidence of right-sided involvement (77%) aligns with established literature on ureteric compression by the gravid uterus, which preferentially affects the right ureter [3]. This asymmetrical involvement further supports the theory that physiological compression remains the dominant factor in anatomical hydroureteronephrosis during pregnancy. Given that physiological compression typically resolves postpartum [13] and can be managed conservatively, the US findings (normal RI, visible ureteral jets) are crucial for avoiding unnecessary intervention.

### Management implications

The detection rate of renal stones in our study (23%) falls within the range reported in the literature [1, 6, 7]. The management outcomes strongly support a conservative and expectant approach for the majority of cases (91%), which is consistent with the understanding that small stones (≤ 7 mm) and physiological hydronephrosis often pass spontaneously or are managed successfully with medical therapy [13, 14]. Surgical intervention was restricted to a small, specific cohort (9%) defined by objective risk factors identified by US (stones > 7 mm, Grade IV hydronephrosis, or complicating renal abscess), allowing for targeted intervention (stenting or percutaneous aspiration) while minimizing fetal risk.

Integration of hydronephrosis grading, RI, ureteric jets, and parenchymal changes allows construction of a management-oriented algorithm. This framework safely identifies patients suitable for conservative therapy and restricts intervention to objectively high-risk cases.

#### Novelty

The algorithm translates imaging into a practical clinical pathway, not merely descriptive data. It answers the critical question: Which patients require immediate intervention vs. expectant management?

#### Clinical implications

Application of this strategy can minimize maternal and fetal risk, reduce unnecessary procedures, and improve resource utilization. The proposed algorithm emphasizes that imaging supports clinical judgment .Surgical interventions, such as ureteric stenting, can cause stent syndrome (urgency, frequency, hematuria), significantly impacting quality of life [15]. By utilizing RI and clinical markers, we avoided these complications in 91% of our cohort.

### Study limitations

- The retrospective nature of this study and lack of formal ROC analysis are noted. Future prospective validation of the RI and clinical algorithm is required
- Interobserver variability of RI not formally assessed
- Lack of advanced statistical modeling

## Conclusion

High-resolution US combined with Doppler RI provides a reproducible, management-oriented approach to acute flank pain in pregnancy. The decision algorithm allows safe conservative management for most patients while identifying those requiring timely intervention.

## Data Availability

All the data generated or analyzed in this study were included in the article, and no further data are recommended to reproduce the results; however, if any further data are needed, they will be available upon reasonable request.
